# Integrated In Vivo and In Vitro Evaluation of a Powder-to-Hydrogel, Film-Forming Polymer Complex Base with Tissue-Protective and Microbiome-Supportive Properties

**DOI:** 10.3390/gels10070447

**Published:** 2024-07-05

**Authors:** Daniel Banov, Guiyun Song, Zahraa Foraida, Oksana Tkachova, Oleksandr Zdoryk, Maria Carvalho

**Affiliations:** 1 Professional Compounding Centers of America (PCCA), Houston, TX 77099, USAmcarvalho@pccarx.com (M.C.); 2 Department of Pharmaceutical Management and Marketing, National University of Pharmacy, 61002 Kharkiv, Ukraine; 3 Department of Pharmaceutical Technologies and Medicines Quality Assurance, National University of Pharmacy, 61002 Kharkiv, Ukraine

**Keywords:** mouse wound healing model, in vitro tissue viability, cell migration assay, microbiome analysis, poloxamer 407, misoprostol, phenytoin, hydrogel, sodium carboxymethyl starch, pullulan, pharmacy compounding

## Abstract

The study aimed to perform a comprehensive in vitro and in vivo evaluation of a newly developed, patent-pending, powder-to-hydrogel, film-forming polymer complex base, which possesses tissue-protective and microbiome-supportive properties, and to compare its characteristics with poloxamer 407. The study used a combination of in vitro assays, including tissue viability and cell migration, and in vivo wound healing evaluations in male diabetic mice. Microbiome dynamics at wound sites were also analyzed. The in vitro assays demonstrated that the polymer complex base was non-cytotoxic and that it enhanced cell migration over poloxamer 407. In vivo, the polymer complex base demonstrated superior wound healing capabilities, particularly in combination with misoprostol and phenytoin, as evidenced by the reduced wound area and inflammation scores. Microbiome analysis revealed favorable shifts in bacterial populations associated with the polymer complex base-treated wounds. The polymer complex base demonstrates clinical significance in wound care, potentially offering improved healing, safety and microbiome support. Its transformative properties and efficacy in drug delivery make it a promising candidate for advanced wound care applications, particularly in chronic wound management.

## 1. Introduction

The increasing prevalence of acute and chronic wounds is a major problem, exacerbated by a number of factors, including an aging population and comorbidities, such as diabetes. A wide variety of dosage forms (creams, gels, ointments, powders, pastes and patches) have been developed for wound management, each designed to address specific aspects of wound treatment, including healing, antimicrobial activity and moisturization [1]. In cases where commercially available pharmaceutical products do not produce the desired outcome, the administration of combination drugs is a valuable therapeutic tool. Physicians often incorporate various active pharmaceutical ingredients (APIs) into compounded medications to optimize wound treatment, such as liothyronine sodium, timolol, phenytoin, misoprostol, lidocaine, gabapentin, ketamine, insulin, arginine, naltrexone, ketoprofen, metronidazole, clindamycin and doxycycline, among others [2,3,4,5]. The efficacy of these medications is dependent on the careful selection of appropriate bases (vehicles) to incorporate the APIs. The critical role of bases is to ensure optimal drug delivery to the target site, thereby facilitating the complex healing process. A notable example is the use of misoprostol in combination with phenytoin in a poloxamer 407 20% gel base. This formulation has been used to enhance the healing of malodorous and/or infected wounds, including pressure ulcers, radiation burns, surgical incisions, oral ulcers and foot lesions [6].

### 1.1. Wound Healing Bases

Wound healing bases may be divided into occlusive bases and semi-occlusive bases. Occlusive bases commonly include ingredients such as gelatine, pectins, carboxymethylcellulose, oils, polyethylene glycol (PEG) and poloxamer 407 gels, whereas semi-occlusive bases typically include oil-in-water emulsions, PEG, silicone, hydrocolloids and hydrogels. Regarding dosage forms, wound care formulations are commonly gels, powders, creams, ointments or liquids [3,4,6,7]. Gel bases are considered promising due to a number of advantages, such as prolonged duration of mucoadhesion, prolonged release of APIs, disruption of biofilm formation and the possibility of maintaining a moist environment. These gel bases often include hydroxypropyl methylcellulose (HPMC), poloxamer 407 gels, PEGs of various weights, silicone, fatty acids and lipids, and may contain pracaxi oil and meadowsweet, as well as mucoadhesive polymer blends. Also widely used are powder bases, such as those prepared with anhydrous polyethylene oxide, mainly because of extended beyond-use dates by default [6].

In the field of modern wound care, the search for innovative solutions has led to the development of novel wound care systems. Recently, attention has turned to film-forming gels that undergo significant compositional change upon application to the skin due to the evaporation of volatile components, resulting in a residual transparent film [8,9]. These films serve a dual purpose when applied to wounds, preventing access to pathogenic bacteria while allowing oxygen and carbon dioxide to permeate. Film-forming gels consist of APIs, solvent systems, polymers and penetration enhancers. The most common film-forming polymers include polyvinyl pyrrolidone, PEG, HPMC, chitosan, polyisobutylene, polyvinyl alcohol, ethyl cellulose, eudragit, acrylate copolymer and polydimethylsiloxane [10,11]. However, the variety of film-forming systems among compounded medications is limited.

Poloxamer 407 is a non-ionic triblock copolymer consisting of a hydrophobic polyoxypropylene core (approximately 56 repeat units) flanked by two hydrophilic polyoxyethylene chains (approximately 101 repeat units each). Poloxamer 407 is widely used in pharmaceutical formulations due to its versatility and advantageous properties [1]. It is used in targeted drug delivery, controlled release systems and as a key component in topical hydrogels. Poloxamer 407 has unique thermosensitive properties that allow it to remain fluid at low temperatures and to form a viscous gel at body temperature, thereby facilitating localized and sustained drug release. Its amphiphilic structure allows solubilization of poorly water-soluble drugs, improving bioavailability. Poloxamer 407’s versatility, biocompatibility and controlled-release capabilities make it a popular choice for compounded medications [6], and it is often used as a comparator.

### 1.2. Newly Developed Polymer Complex Base

**Figure 1:**
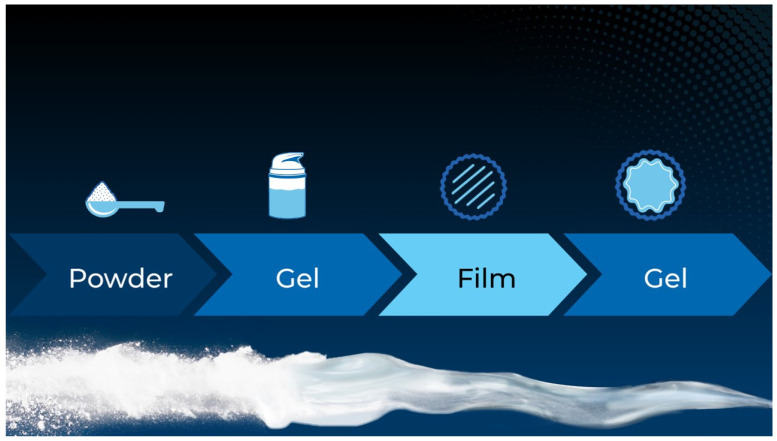
Sequential transformation scheme of the PCB (powder into hydrogel, hydrogel into film, film into hydrogel).

A patent-pending, powder-to-hydrogel, film-forming polymer complex base (PCB), which possesses tissue-protective and microbiome-supportive properties, has been recently developed. This new base is transformative in nature, changing from a powder to a hydrogel upon wetting and then forming a protective film that adheres seamlessly to the wound (Figure 1) [12]. The key feature of the PCB that differentiates it from others is its ability to revert to a hydrogel state upon remoistening, which is a distinct advantage in wound care applications. The composition of this base is unique and includes ectoin, trehalose, β-cyclodextrin, sodium carboxymethyl starch, inulin and pullulan.

Ectoin assumes the role of a DNA protector against a number of environmental stresses, ranging from biotic to abiotic factors, including UV light and various forms of radiation [13]. It serves as a stabilizer for intracellular macromolecules, enzymes, nucleic acids and various proteins. Ectoin increases cellular hydration and maintains turgor under adverse conditions [14].

Trehalose plays a key role in promoting the extensive spreading of the epidermal layer and shows a remarkable ability to significantly accelerate wound closure. Furthermore, its influence extends to angiogenesis, as the application of trehalose leads to a remarkable increase in capillary formation [15]. Trehalose also shows reparative effects on cells damaged by UV irradiation and radiation, particularly in the case of keratinocytes [16]. This reparative property is complemented by its ability to prolong cell viability and water retention time, providing cellular protection under conditions of high environmental desiccation [17].

Cyclodextrins are increasingly used in the development of innovative therapies [18]; their derivatives serve as versatile host macromolecules, forming supramolecular compounds through host–guest recognition, and as common drug carriers for the encapsulation of various substances. This allows the design of optimal compositions by combining hydrogels, with cyclodextrin hydrogels providing a moist environment essential for wound healing [19].

Carboxymethyl starch is another component of the hydrogel, and it is often chosen for its physical and mechanical properties. The combination of carboxymethyl starch with other polymers results in a hydrogel that is characterized by self-recoverable extensibility–compressibility, biodegradability and transparency, making it a suitable candidate for use in wound healing applications [20,21].

Inulin represents a promising advancement in wound care due to its probiotic, hydrophilic, biodegradable, biocompatible and non-toxic properties [22]. The ability of inulin to support cell growth and tissue regeneration has been documented [23]. In addition, its effect on wound healing extends to the regulation of oxidative and inflammatory responses. Topical application of inulin shows enhancement of keratinocyte migration, acceleration of re-epithelialization and an increase in fibroblast response to skin wounds. The physicochemical properties of inulin, as well as pullulan, in combination with other polymers allow the formation of a protective film.

Pullulan-based hydrogels are recognized for their superior capabilities in drug delivery systems. Pullulan is known for its ability to accelerate the processes of fibroblast proliferation, collagen production and maturation, and epithelialization [24].

### 1.3. In Vivo and In Vitro Methods

Thorough evaluation of topical wound healing products requires the application of both in vivo and in vitro methods, each of which plays an important role in assessing the overall performance and therapeutic efficacy of these products [25]. In vitro methods are often used for preliminary analysis due to their effectiveness in predicting wound healing potential. These methods are also consistent with the three Rs (Replacement, Reduction and Refinement), which advocate more humane approaches to animal research. In vitro analysis involves the study of wound-healing-relevant cell cultures, such as fibroblasts and keratinocytes, which are treated with the formulation under investigation [26]. These studies evaluate key parameters, such as cell viability, proliferation, migration and gene expression, and provide fundamental insight into the healing properties of formulations [25,27]. When formulations have antimicrobial properties, in vitro testing is performed against relevant pathogens using methods such as zone-of-inhibition (ZOI) and dilution techniques to determine antimicrobial efficacy [25,28]. For topical drug delivery products, studies are conducted to analyze the controlled release profiles of therapeutic agents to ensure their effective delivery over time [25,29].

In vivo methods traditionally involve animal studies using laboratory mice or rats with artificially induced wounds. These methods are invaluable for predicting healing effects by examining parameters such as wound closure, inflammation and tissue regeneration [30]. Tissue samples from treated wounds are analyzed histologically to observe changes in tissue structure, inflammatory response and the healing process [25,30]. The external appearance of a wound, including size reduction and visual signs of healing, is monitored over time using a variety of qualitative and quantitative methods to assess the healing progress (macroscopic observation) [31,32]. Together, in vivo and in vitro methods provide a comprehensive understanding of the mechanisms of action, efficacy and safety of topical wound healing formulations.

The primary objective of this study was to perform a comprehensive in vitro and in vivo evaluation of a newly developed, patent-pending, powder-to-hydrogel, film-forming PCB, which possesses tissue-protective and microbiome-supportive properties, and to compare its characteristics with the widely used standard, poloxamer 407.

## 2. Results

### 2.1. In Vitro Tissue Viability Assay

**Figure 2:**
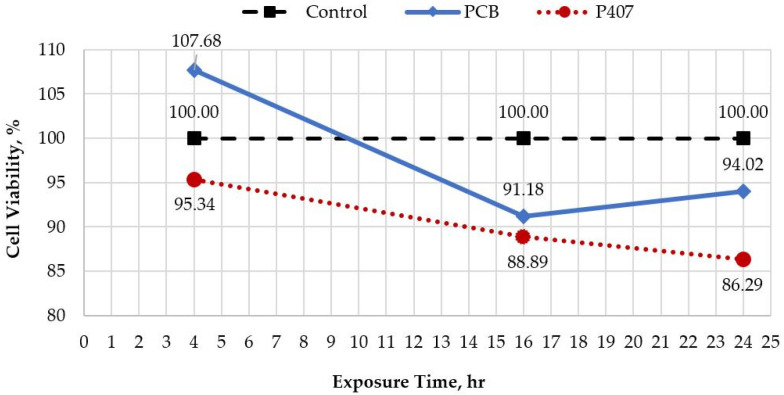
Effects of PCB and P407 on skin cell viability and irritancy. (The solid blue line shows the change in cell viability of the PCB-treated samples; the dashed black line shows the cell viability of the control samples; the dashed red line shows the cell viability of the P407-treated samples).

Cytotoxicity evaluations were performed in the EpiDerm skin model to evaluate the non-toxic nature of the PCB and P407. Cell viability results for the negative control group remained at 100%. Cell samples treated with the PCB and P407 showed viability decreases of 91.18 ± 1.99% and 88.89 ± 1.40% at 16 h and 94.02 ± 2.42% and 86.29 ± 2.63% at 24 h, respectively (Figure 2). As shown in Figure 2, none of the samples reached the ET50 value at 24 h, with all results remaining above 85%.

### 2.2. In Vitro Cell Migration Assay

**Figure 3:**
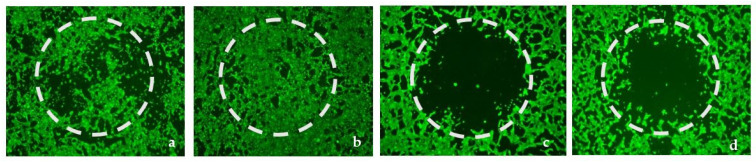
Microscopic visualization of keratinocyte migration (green fluorescence) 24 h after application: (**a**) PCB; (**b**) phenytoin and misoprostol in the PCB; (**c**) P407; (**d**) phenytoin and misoprostol in P407. The white dotted circles indicate the areas where cells migrated.

**Figure 4:**
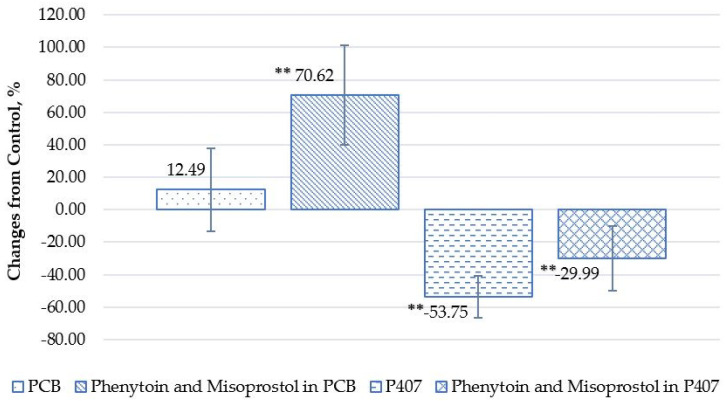
Mean percentage change in cell migration in the presence of phenytoin and misoprostol topical hydrogel in the PCB and P407, from control following 24 h post-application. (Notes: Statistical analysis performed using independent-samples *t*-tests with comparison to the control group. **: *p* < 0.01).

After 24 h of treatment with the test products, fluorescence data revealed remarkable effects on cell migration. PCB alone and in combination with misoprostol and phenytoin increased cell migration by 12.49 ± 25.56% and 70.62 ± 43.27%, respectively, when compared to untreated cells. Conversely, P407 did not increase cell migration, resulting in a negative mean change. The mean change for P407 alone was 53.75 ± 12.90%, and for the combination with misoprostol and phenytoin it was 29.99 ± 22.85% (Figure 3 and Figure 4).

### 2.3. In Vivo Evaluation of Wound Healing

A comparison of the relative wound areas of the mice in the five groups, G1–G5, at three time intervals (days 3, 9 and 15) is shown in Table 1.

The data include means, standard deviations (SDs) and *p*-values. For group G1, there were no *p*-values at all time intervals (days 3, 9 and 15) because it was the control group. For group G2, a statistically significant reduction in wound area of almost 60% was observed at day 9 (*p*-value = 0.004), and almost complete wound healing was observed at day 15. In group G3, the wound area was halved on day 9, and complete wound healing was observed on day 15 (*p*-value = 0.07). Groups G4 and G5 also showed almost complete healing on day 15.

**Figure 5:**
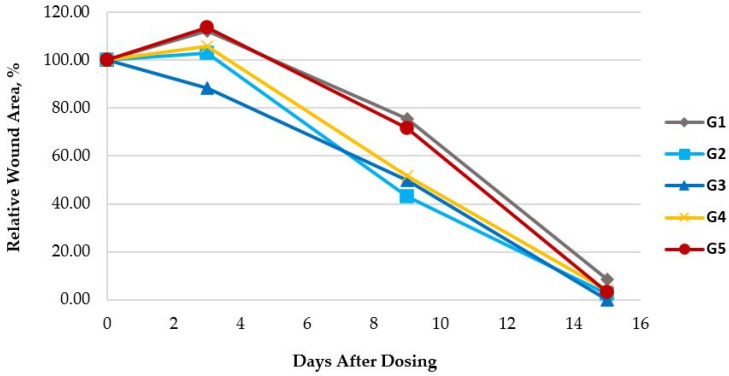
Mean percentage of relative wound area for the mice in the control and G2-G5 groups, for a total of 15 days. (The grey line (G1) represents the control group’s relative wound area; the light-blue line denotes the relative wound area of the G2 group, treated with PCB; the dark-blue line denotes the relative wound area of the G3 group, treated with phenytoin/misoprostol in PCB; the yellow line denotes the relative wound area of the G4 group, treated with P407; and the red line denotes the relative wound area of the G5 group, treated with phenytoin/misoprostol in P407).

A detailed comparative analysis of the change in total wound area for the mouse groups G1–G5 is shown in Figure 5.

**Figure 6:**
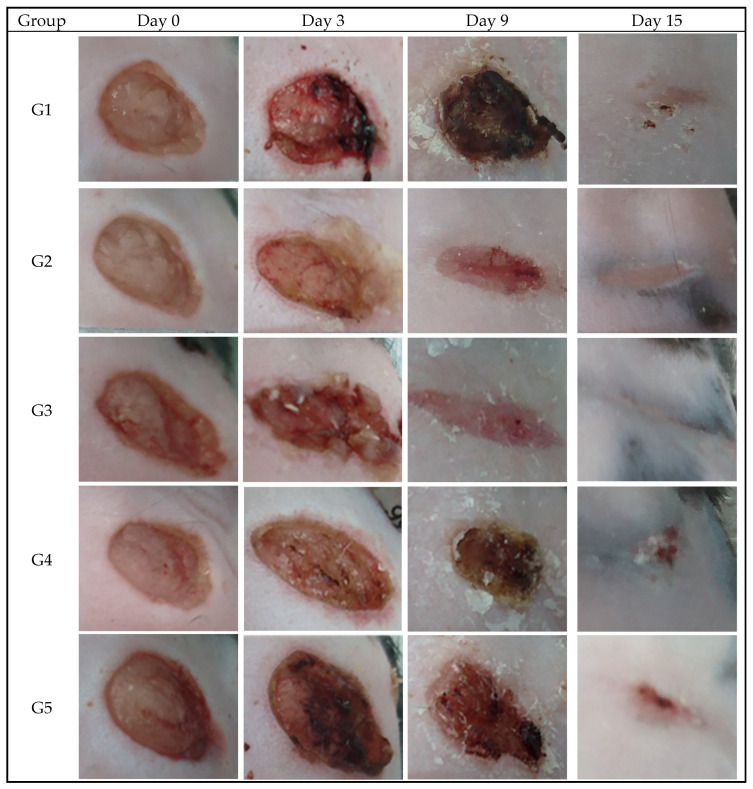
Digital photographs of the wound areas for selected mice in the control group and the test groups, G2–G5, at day 0 (before treatment) and days 3, 9 and 15 of treatment. (The images display the wound areas of selected mice captured on day 0 (pre-treatment) and on days 3, 9 and 15 during treatment. G1 represents the control group; G2 was treated with PCB; G3 was treated with phenytoin/misoprostol in PCB; G4 was treated with P407; G5 was treated with phenytoin/misoprostol in P407).

Figure 5 shows that on day 3 the relative wound areas treated with the PCB and phenytoin/misoprostol in PCB were 10–20% smaller than those of the control group and the G4 group treated with P407. A comparable result was observed on day 9. The difference between the wounds of groups G3 and G5 on days 3 and 9 was 25.28% (*p*-value = 0.02) and 21.87% (*p*-value = 0.08), respectively, indicating a significant difference in results between these two groups. It is important to note that by day 15 the wounds were almost closed in all groups, with the exception of the control group. The mean percentage results obtained are consistent with the external appearances of the wounds during the study, which were documented photographically. Examples of photographs of wounds from selected mice are shown in Figure 6.

**Figure 7:**
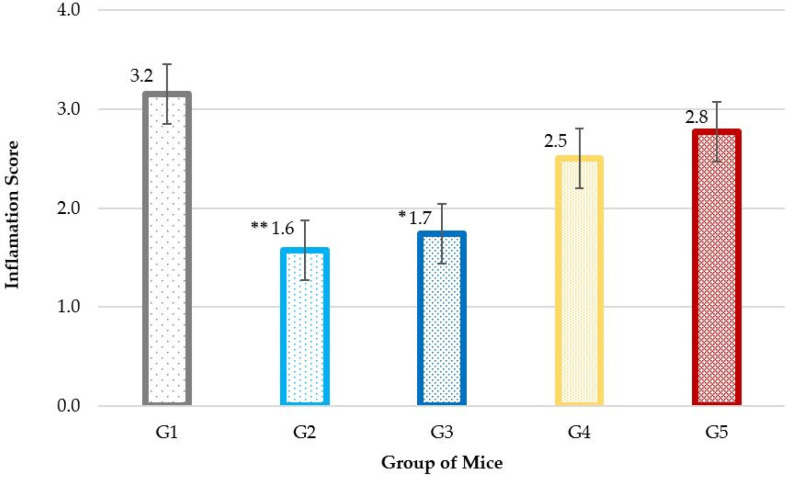
Inflammation score across mouse groups. The chart illustrates the inflammation scores of the different groups: G1 is the control group; G2 was treated with PCB; G3 was treated with phenytoin/misoprostol in PCB; G4 was treated with P407; G5 was treated with phenytoin/misoprostol in P407. (Notes: Statistical analysis was performed using independent-samples *t*-test with comparison to the control group. *: *p* < 0.05; **: *p* < 0.01).

The ISs for the skin of the mice in the various groups are depicted in Figure 7. At the endpoint of the experiment, the results showed that the G2 and G3 groups had a significant reduction in IS (*p* < 0.01) compared to the control group, G1. No significant differences were observed among groups G1, G4 and G5.

### 2.4. Microbiome Analysis

Microbiome analysis of mouse wound sites revealed dynamic changes over a 15-day period (Table 2).

**Figure 8:**
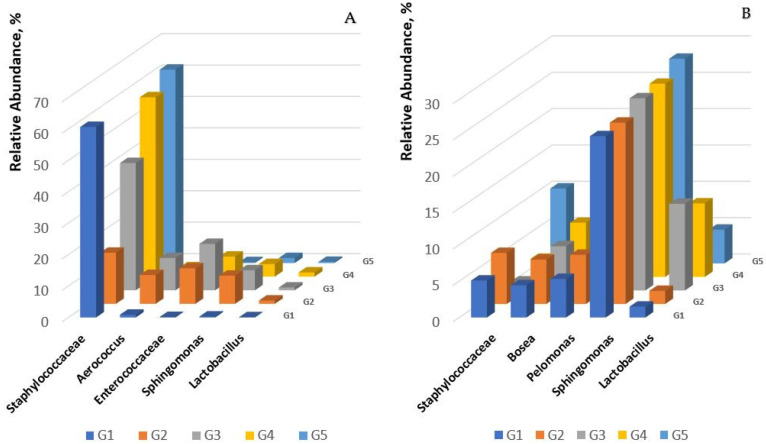
Genus-level microbial composition analysis on days 7 and 15. (**A**) Distribution of predominant microorganisms in the microbiome on day 7. (**B**) Distribution of predominant microorganisms in the microbiome on day 15. (The groups represented are as follows: G1—control group; G2—treated with PCB; G3—treated with phenytoin/misoprostol in PCB; G4—treated with P407; G5—treated with phenytoin/misoprostol in P407).

On day 0, *Aerococcus* was the predominant genus in groups G1 to G5, typically ranging from 22% to 52% abundance, while *Sphingomonas* (8–16%) and *Lactobacillus* (1–5%) were consistently observed in all the groups, with the abundance of *Trabulsiella* being significantly higher in G1 and G5. By day 7, there was a significant shift towards proliferation of *Staphylococcaceae* in groups G1, G4 and G5, with levels around 60%. In contrast, this genus represented only 16.26% in group G2 and 40.49% in G3. At the same time, *Enterococcaceae* spiked in groups G2 and G3, indicating an altered microbial landscape, possibly influenced by the use of the test products. Figure 8 shows the distribution of predominant genera on day 7. By day 15, the presence of *Sphingomonas* had increased uniformly across all groups to 26–28% (Figure 8). Collectively, all these species, except Staphylococcus species, are considered normal flora for mice, indicating that groups 2 and 3 favored and supported normal flora over the pathogenic species.

Conversely, the levels of *Staphylococcaceae* decreased dramatically in groups G3 and G4, and to a lesser extent in G2, which may be related to the wound healing and normal-flora-supportive effects observed in these groups. In addition, there was an increase in *Lactobacillus* in groups G3 and G4 by day 15. This trend was inversely related to the reduction in *Staphylococcaceae* in these groups.

## 3. Discussion

### 3.1. In Vitro Tissue Viability Assay

The ET50 values for both PCB and P407 exceeded 24 h, indicating a favorable safety and toxicological profile for both bases. For P407, the cell viability results of over 85% for 24 h post-treatment are consistent with findings reported in studies involving MTT assays for keratinocytes [33] and stem cells [34]. These results are consistent with general guidelines that products with an ET50 of 24 h (or greater) are expected to be non-toxic to human skin [35]. Meanwhile, the PCB showed slightly higher cell viability results compared to P407. The observed increase in cell viability from 91.18% at 16 h to 94.02% at 24 h could indicate a potential wound healing effect of the PCB, which may also contribute to an increase in cell proliferation.

The tissue-protective properties of PCB are primarily attributed to its diverse composition and coincide with the data from the literature review. Ectoin and trehalose provide stabilization of cell membranes and proteins, protecting tissues from dehydration and environmental stress [13,14,15]. Sodium carboxymethyl starch and pullulan provide a film-forming effect that further protects the wound area from external contaminants and mechanical damage [20,21,22,23].

### 3.2. In Vitro Cell Migration Assay

The findings regarding the influence of P407 on cell migration activity are consistent with results reported in the literature. Specifically, P407 hydrogels incorporating components such as alginic acid and carboxymethylcellulose have been shown to reduce cell migration in studies using mouse fibroblast cells [36]. In addition, scratch wound healing and migration assays conducted on human keratinocytes and melanoma cells have highlighted the ability of a gallic acid-infused P407 gel to inhibit cell migration [37]. The comparison of keratinocyte migration in vitro suggests a pronounced promoting effect of the PCB on the migration of HOK. This indicates potentially more favorable conditions for wound healing compared to P407. In addition, the phenytoin/misoprostol in PCB stimulated cell migration more intensely. Keratinocyte migration is part of the re-epithelialization process in wound healing, and therefore the PCB is likely to have greater wound healing abilities than the corresponding base P407. Such accelerated cell migration should lead to faster wound closure and healing in vivo. These results underscore the potential of the PCB, especially phenytoin/misoprostol in PCB, to enhance the wound healing process, which is key in clinical practice.

### 3.3. In Vivo Evaluation of Wound Healing

The in vivo wound healing evaluation provided compelling evidence of the efficacy of the PCB and misoprostol/phenytoin in PCB over the 15-day period. Group G4 (treated with P407) also exhibited effective wound healing, which was particularly noticeable by day 9. The groups G1 (control) and G5 (treated with phenytoin/misoprostol in P407) displayed the least change in wound area over time. *p*-value analysis indicates that the most significant departures from the control were observed in groups G2 and G4, on day 9. The in vivo evaluation showed that PCB significantly improved wound healing compared to the control and P407 groups. This effect may be attributed to the unique properties of PCB, such as its ability to form a protective film and its bioactive components, which are believed to promote faster re-epithelialization and reduce inflammation. The superior wound closure observed in the PCB-treated groups suggests enhanced tissue regeneration. The film formed by PCB not only protects the wound from external contaminants, but also maintains a moist environment conducive to healing.

### 3.4. Microbiome Analysis

Microbiome analysis adds another layer of understanding to the wound healing process. The initial abundance of *Aerococcus* and the subsequent proliferation of *Staphylococcaceae* and *Enterococcaceae* are consistent with known bacterial behaviors at wound sites. However, the marked decrease in *Staphylococcaceae* by day 15 in groups G2 and G3, which showed the best healing, suggests a possible inhibitory effect of the test products on this genus of microorganisms, which is often associated with wound infection. The increased presence of *Lactobacillus* in the later stages of healing in the most responsive groups (G3 and G4) suggests its potential role in promoting wound closure. This is consistent with literature supporting the beneficial effects of *Lactobacillus* in wound management due to its ability to modulate the immune response and inhibit pathogen colonization [38], including diabetic wounds [39].

The microbiome-supporting properties of PCB are explained by the presence of inulin and trehalose, prebiotics that promote the growth of beneficial microorganisms on the skin. These help maintain a healthy microbiome balance, which is critical for preventing infection and supporting the natural healing process [22,23]. The protective film formed by the components shields the wound from further injury and infection, while the stabilizing and prebiotic effects provide a supportive environment for tissue regeneration.

The results of the in vitro and in vivo studies complement each other regarding the efficacy of the PCB and misoprostol/phenytoin in PCB. In vitro assays demonstrated that both the PCB and the misoprostol/phenytoin in PCB are non-cytotoxic, with potential to facilitate wound healing. This is consistent with the in vivo results, where improved wound healing, reduced inflammation and favorable microbiome changes were observed, particularly in the groups treated with the PCB and the misoprostol/phenytoin in PCB. The cell migration assay results for P407 and the corresponding compounded medication correlated with the in vivo wound healing results in terms of healing rate, when compared to the PCB. This conformity between in vitro predictability and in vivo results underscores the reliability of the chosen test methods and the potential translational applicability of the PCB compounded medications in wound care.

The promising results from both in vitro and in vivo evaluations open avenues for clinical applications of personalized PCB compounded medications. The efficacy in accelerating wound healing, closing the wound with a film, reducing bacterial contamination in and around the wound, and modulating the wound microbiome suggests potential for the development of individualized wound care treatments, particularly for chronic or non-healing wounds [2]. The PCB is well suited for individualized treatment of wound processes, as it allows for variation in several factors from the choice of dosage form (powder or gel) and application method, which can be tailored to the wound type and the patient’s preferences, to the combination with different APIs according to the patient’s needs and the physician’s treatment strategy [6]. The ability to effectively and safely deliver active ingredients, as demonstrated in this study, is particularly important in the context of antibiotic delivery and the need for targeted microbial control in wound management.

## 4. Conclusions

The in vitro tissue viability assay confirmed that the PCB is non-cytotoxic. This was evidenced by the sustained cell viability of over 85% after 24 h, ensuring safety for clinical use. The PCB, alone and in combination with misoprostol and phenytoin, demonstrated an increase in keratinocyte migration, which is essential for wound re-epithelialization. This ability is critical for accelerated wound closure and healing, potentially positioning the PCB as a superior alternative to the standard P407 base.

The in vivo evaluations showed improvements in wound healing with PCB, alone and in combination with misoprostol and phenytoin. In particular, there was an evident reduction in wound area and inflammation and faster healing, especially with the PCB compounded medication containing misoprostol and phenytoin. The microbiome study of the wound sites also showed a reduction in staphylococcal infection, which is commonly associated with biofilm formation, as compared to the control and comparator groups, which potentially correlates with better healing outcomes and creates a favorable microbial environment for wound healing.

The conformity between the in vitro predictability and in vivo results underscores the reliability and translational applicability of the chosen test methods. This study paves the way for the clinical application of PCB personalized compounded medications and offers a novel and potentially more effective solution for wound management and care.

## 5. Materials and Methods

This study evaluated a newly developed, patent-pending, powder-to-hydrogel, film-forming polymer complex base (trade name: EctoSeal P2G), hereinafter referred to as PCB. Poloxamer 407 NF gel 20% (trade name: Pluronic^®^ F-127), which is known for its thermosensitive drug delivery properties and facilitation of sustained drug release, was used in this study for comparison purposes. Poloxamer is hereinafter referred to as P407. APIs, bases and compounded medications were prepared and supplied by PCCA (Professional Compounding Centers of America, Houston, TX, USA): EctoSeal P2G (lot 9515743), Poloxamer 407 NF (lot C201024), Phenytoin (lot C198892) and Misoprostol (lot C202695).

The compounded medications assessed in this study were (1) phenytoin 2% and misoprostol 0.0024% topical hydrogel in 20% PCB and (2) phenytoin 2% and misoprostol 0.0024% topical hydrogel in 20% P407. The PCB formulations were prepared by blending 2% phenytoin, 0.0024% misoprostol, 20% PCB powder, 1.5% benzyl alcohol, 10% PEG 300 and purified water in an electronic mortar and pestle for three minutes at setting 2. The mixture was then milled once to refine the particle size and texture. The P407 formulations were prepared by the same method, substituting the PCB with pre-made 20% P407. The bases and formulas were stored in a refrigerator at 5 ± 3 °C to maintain their stability and efficacy throughout the study period.

### 5.1. In Vitro Tissue Viability Assay

The three-dimensional in vitro human EpiDerm system (EPI-200) (lot 39169) (MatTek, Ashland, OR, USA), a model of human epidermis, and the MTT-100 Kit were used for the assay. The preparation, dosing and extraction of EpiDerm tissues and the use of the MTT-100 Kit were carried out strictly following the manufacturer’s protocol [35]. Each EpiDerm tissue sample was treated in triplicate with 100 μL of two different bases: PCB 20% and P407 20% (comparator). A separate set of EpiDerm tissues was left untreated to serve as a negative control group. The treated and control EpiDerm tissues were exposed to their respective conditions for three time periods: 4, 16 and 24 h. After each exposure period, the dosing solutions were carefully removed from the tissues. The EpiDerm cells were then analyzed for cell viability using the 3-(4,5-dimethylthiazol-2-yl)-2,5-diphenyltetrazolium bromide (MTT) ET50 assay. This method relies on the conversion of the yellow tetrazolium salt MTT, which can permeate cell membranes, into blue/purple formazan crystals by cells that are metabolically active. Sample absorbance was read at 570 nm and the reference absorbance at 650 nm using a CLARIOstar plate reader (BMG LABTECH GmbH, Ortenberg, Germany). The % viability of each sample was calculated using the following formula:

$$iability,\%=\frac{OD\left(sample\right)}{OD\left(negative\;control\right)}\times 100\%$$

where OD (sample) stands for the optical density of the sample and OD (negative control) stands for the optical density of the sample of the negative control. The data obtained were analyzed using Microsoft Excel (Microsoft 365; Microsoft, Washington, DC, USA).

### 5.2. In Vitro Cell Migration Assay

To evaluate the effect of the PCB on human oral keratinocyte (HOK) migration from the edge to the center of the wound, an in vitro evaluation was performed using the Oris™ cell migration assay kit (Platypus Technologies, LLC, Fitchburg, MA, USA) according to established procedures [40]. The test formulations selected for this study were phenytoin 2% and misoprostol 0.0024% topical hydrogel in the PCB and phenytoin 2% and misoprostol 0.0024% topical hydrogel in P407. For comparison purposes, the bases were tested alone and with APIs.

Cell culture: HOKs (ScienCell Research Laboratories, San Diego, CA, USA) were grown in T-75 tissue culture flasks from a cryopreserved state to 70–80% confluence. At the desired confluence, the cells were trypsinized, counted and resuspended in growth medium. The cell suspension was seeded into 6-well plates at a final density of 20,000 cells per well. Care was taken to add 100 µL of cell suspension to each well without disturbing the cell seeding stoppers. The plates were then incubated at 37 °C in a 5% CO_2_ atmosphere until the cells reached 90–95% confluence.

Cell migration assay: PCB, P407 and their corresponding compounded medications were accurately weighed to prepare a 5% stock solution in growth medium, followed by thorough vortexing. Subsequent dilutions were made to obtain 0.01% *v*/*v* solutions. After overnight incubation, the stoppers were carefully removed, leaving the reference wells intact, and the wells were then gently washed with media to remove nonadherent cells. Fresh culture media containing the diluted test samples were added to the wells. The cells were incubated for an additional 24 h at 37 °C and 5% CO_2_ to facilitate migration. After migration, the cells were stained with Calcein AM (Gibco/Life Technologies, Carlsbad, CA, USA), and the wells were rinsed with 100 µL Dulbecco’s phosphate-buffered saline (D-PBS) before and after the staining.

Data collection and processing: Fluorescence detection was performed using a CLARIOstar plate reader (BMG LABTECH GmbH, Ortenberg, Germany) equipped with Stars software (V6.20 Edition 3) for analysis using 483/14 nm excitation and 530/30 nm emission filters. The plate reader quantified the fluorescence signal and recorded the raw RFU values. These values were then exported to Excel, where the average RFU for each well was calculated to quantify cell migration (percentage change from control). In addition, a TS100-F inverted microscope (Nikon, Tokyo, Japan) was used to visually document the cell migration process.

### 5.3. In Vivo Evaluation of Wound Healing

The in vivo evaluation test was performed by GemPharmatech Co., Ltd. (San Diego, CA, USA), which is accredited by the International Laboratory Animal Evaluation and Accreditation Management Committee (AAALAC International) and has an animal use license approved by the Animal Management Committee of the Jiangsu Provincial Department of Science and Technology (certification number: 511214900020707; animal protocol: GPTAP20230810-4; project number: PO-GJC052023078305-01). The protocol and all modifications or procedures related to the care and use of the animals in this study were reviewed and approved by the Institutional Animal Care and Use Committee (IACUC) of GemPharmatech Co., Ltd. before the study was conducted. Animal care and use were conducted in accordance with AAALAC guidelines.

The in vivo study was designed to evaluate the effects of PCB and P407, as well as the corresponding compounded medications, including phenytoin 2% and misoprostol 0.0024%.

Experimental design: Diabetic male mice of the C57BL/6J/BKS-db strain (Mus musculus, certificate numbers: 511214900020707 and 511214900021251), microbial-grade SPF, aged 8–10 weeks, were used in this study. The mice (n = 20) were divided into 5 groups (n = 4 each) according to blood glucose level and body weight: a control group (G1) and experimental groups G2 (treated with PCB), G3 (treated with phenytoin/misoprostol in PCB), G4 (treated with P407) and G5 (treated with phenytoin/misoprostol in P407). All mice were anesthetized, and their dorsal hair was shaved with a safety blade. Two full-thickness excisional skin wounds were made on the back of each mouse using an 8 mm biopsy punch. Images of each wound were taken on days 0, 3, 9 and 15, and the wound area was measured. Subsequently, 300 mg/mouse of the test product was applied daily to the skin wounds of the mice in groups G2, G3, G4 and G5 for a total of 15 days. Wound areas were measured, normalized as a percentage of day 0 and expressed as means and standard deviations (means ± SDs).

Inflammation scores (ISs): The skin tissues were collected on day 16, at the endpoint of the experiment. The ISs were determined from the samples by evaluating potential histologic changes in the skin of the mice using hematoxylin and eosin staining. The structural changes of the skin were scored on a 4-point scale: 1—lesions are barely visible; 2—lesions are easily visible with few/small foci or areas involved; 3—lesions are clearly present and noticeable in size and/or number; 4—lesions are present in large numbers and/or prominent in size in many parts of the tissue section.

### 5.4. Microbiome Analysis

For microbiome sequencing, peri-wound microbiome samples were collected with a swab on days 0, 7 and 15. Swabs were taken from the skin around the wound within a radius of no more than 1.5 cm^2^, which was marked with a marker from the beginning to the end of the study. Taxonomic composition microbiome analysis was performed with QIIME 2 2019.4 [41] with slight modifications, according to the official tutorials [42].

### 5.5. Statistical Analysis

Statistical analysis was performed using independent-samples *t*-tests to compare the treatment groups. *p*-values less than 0.05 were considered statistically significant. Data were expressed as means ± standard deviations (SDs). All statistical analyses were conducted using Microsoft Excel (Version 2205; Microsoft, USA).

## Figures and Tables

**Table 1:** Comparative analysis of relative wound area measurements, with reference to day 0, across treatment groups.

Group	Day	Relative Wound Area		
		Mean	SD	*p*-Value
G1	D3	112.36	26.48	-
	D9	75.44	24.30	-
	D15	8.36	8.35	-
G2	D3	103.02	14.18	0.39
	D9	42.93	11.97	0.004 **
	D15	2.29	4.45	0.09
G3	D3	88.45	15.89	0.06
	D9	49.67	22.66	0.07
	D15	0.00	0.00	0.03
G4	D3	105.66	8.16	0.50
	D9	51.48	15.58	0.03 *
	D15	3.79	5.48	0.22
G5	D3	113.73	19.76	0.91
	D9	71.54	19.59	0.73
	D15	2.99	5.54	0.15

Notes: G1 (control), G2 (treated with PCB), G3 (treated with phenytoin/misoprostol in PCB), G4 (treated with P407) and G5 (treated with phenytoin/misoprostol in P407). Statistical analysis performed using independent-samples *t*-tests with comparison to G1. *: *p* < 0.05; **: *p* < 0.01.

**Table 2:** Comparative genus-level microbial distribution of wound healing over 15 days.

Group	*Day*	*Aerococcus*	*Sphingomonas*	*Trabulsiella*	*Lactobacillus*	*Staphylococcaceae*	*Enterococcaceae*
G1	D0	35.80 ± 12.26	12.30 ± 8.30	6.55 ± 13.10	4.58 ± 1.69	2.11 ± 1.43	0.56 ± 0.94
	D7	0.89 ± 1.53	0.16 ± 0.23	6.61 ± 11.52	0.10 ± 0.09	60.64 ± 24.80	0.29 ± 0.22
	D15	1.60 ± 3.02	24.95 ± 14.17	<0.01	1.50 ± 1.41	15.09 ± 10.65	<0.60
G2	D0	33.58 ± 33.80	8.50 ± 7.24	3.36 ± 4.36	5.80 ± 5.55	3.52 ± 5.12	0.07 ± 1.25
	D7	9.20 ± 9.72	8.99 ± 3.75	1.67 ± 2.57	1.07 ± 0.60	16.28 ± 18.84	11.32 ± 20.30
	D15	1.08 ± 2.15	24.95 ± 15.53	<0.01	1.81 ± 1.48	7.02 ± 10.35	<0.60
G3	D0	51.46 ± 23.22	8.45 ± 4.20	0.18 ± 1.83	3.36 ± 1.97	2.48 ± 1.54	1.03 ± 0.55
	D7	10.26 ± 12.05	6.39 ± 5.76	0.04 ± 0.41	0.87 ± 0.61	40.49 ± 19.26	14.77 ± 18.72
	D15	1.26 ± 1.12	26.44 ± 18.97	<0.01	11.93 ± 13.42	1.24 ± 1.15	<0.60
G4	D0	22.87 ± 3.33	16.52 ± 9.31	1.29 ± 2.10	4.66 ± 0.98	2.97 ± 2.00	1.75 ± 2.34
	D7	2.99 ± 3.51	4.03 ± 7.28	1.02 ± 2.04	1.34 ± 1.83	57.09 ± 45.31	1.08 ± 1.00
	D15	0.94 ± 0.42	26.58 ± 9.69	<0.01	10.12 ± 9.79	2.50 ± 1.20	<0.60
G5	D0	40.32 ± 16.41	12.37 ± 8.17	8.57 ± 16.09	1.29 ± 0.56	0.70 ± 0.82	0.63 ± 1.37
	D7	5.15 ± 7.88	1.61 ± 1.63	0.04 ± 0.17	0.38 ± 0.30	61.56 ± 40.27	0.11 ± 0.06
	D15	3.61 ± 3.95	28.16 ± 6.33	<0.01	4.65 ± 2.82	10.31 ± 10.37	<0.60

Notes: G1 (control), G2 (treated with PCB), G3 (treated with phenytoin/misoprostol in PCB), G4 (treated with P407) and G5 (treated with phenytoin/misoprostol in P407).

## Data Availability

No new data were generated in the study.
